# Supplementary material for: A single inactivating amino acid change in the SARS-CoV-2 NSP3 Mac1 domain attenuates viral replication in vivo
Source: PLoS Pathog. 2023 Aug 31;19(8):e1011614. doi: 10.1371/journal.ppat.1011614 (PMC10499221; doi:10.1371/journal.ppat.1011614)
Supplement: S1 Table — (DOCX) [file ppat.1011614.s011.docx]

**S1 Table.** RT-qPCR primers utilized in this study

| **Primer Name** | **Primer Sequence (5’ 🡪 3’)** |
| --- | --- |
| SARS2_N_F | AAATTTTGGGGACCAGGAAC |
| SARS2_N_R | TGGCACCTGTGTAGGTCAAC |
| h18S_F | AACCCGTTGAACCCCATT |
| h18S_R | CCATCCAATCGGTAGTAGCG |
| hGAPDH_F | GGAGCGAGATCCCTCCAAAAT |
| hGAPDH_R | GGCTGTTGTCATACTTCTCATGG |
| hISG15_F | CTGTTCTGGCTGACCTTCG |
| hISG15_R | GGCTTGAGGCCGTACTCC |
| hIFNb_F | CAGGAGAGCAATTTGGAGGA |
| hIFNb_R | CTTTCGAAGCCTTTGCTCTG |
| hIL6_F | AGGAGACTTGCCTGGTGAAA |
| hIL6_R | GCTCTGGCTTGTTCCTCACT |
| hSTAT1_F | CAGCTTGACTCAAAATTCCTGGA |
| hSTAT1_R | TGAAGATTACGCTTGCTTTTCCT |
| hIFNa_F | GACTCCATCTTGGCTGTGA |
| hIFNa_R | TGATTTCTGCTCTGACAACCT |
| hMx1_F | GGTGGTCCCCAGTAATGTGG |
| hMx1_R | CGTCAAGATTCCGATGGTCCT |
| mGAPDH_F | AGGTCGGTGTGAACGGATTTG |
| mGAPDH_R | TGTAGACCATGTAGTTGAGGTCA |
| mIFNa4_F | TCCATCAGCAGCTCAATGAC |
| mIFNa4_R | AGGAAGAGAGGGCTCTCCAG |
| mMX1_F | GACCATAGGGGTCTTGACCAA |
| mMX1_R | AGACTTGCTCTTTCTGAAAAGCC |
| mISG15_F | GGCCACAGCAACATCTATGA |
| mISG15_R | CGCAAATGCTTGATCACTGT |
| mIFNB1_F | TCAGAATGAGTGGTGGTTGC |
| mIFNB1_R | GACCTTTCAAATGCAGTAGATTCA |
| mIL6_F | GAGGATACCACTCCCAACAGACC |
| mIL6_R | AAGTGCATCATCGTTGTTCATACA |
| mTNFα_F | GAACTGGCAGAAGAGGCACT |
| mTNFα_R | AGGGTCTGGGCCATAGAACT |
| mCXCR4_F | GACTGGCATAGTCGGCAATG |
| mCXCR4_R | AGAAGGGGAGTGTGATGACAAA |
| mCCL2_F | CTTCTGGGCCTGCTGTTCA |
| mCCL2_R | CCAGCCTACTCATTGGGATCA |
