# Supplementary material for: A single inactivating amino acid change in the SARS-CoV-2 NSP3 Mac1 domain attenuates viral replication in vivo
Source: PLoS Pathog. 2023 Aug 31;19(8):e1011614. doi: 10.1371/journal.ppat.1011614 (PMC10499221; doi:10.1371/journal.ppat.1011614)
Supplement: S2 Table — (DOCX) [file ppat.1011614.s012.docx]

**S2 Table.** Data collection and refinement statistics for X-ray crystal structures reported in this work.

|  | **Mac1 N40D** | **Mac1 N40D + ADP-ribose** |
| --- | --- | --- |
| PDB code | 8SH6 | 8SH8 |
| **Experimental details** | | |
| Beamline | ALS 8.3.1 | ALS 8.3.1 |
| Temperature (K) | 100 | 100 |
| Transmission (%) | 100 | 100 |
| Wavelength (Å) | 0.7749 | 0.7749 |
| Energy (keV) | 16 | 16 |
| No. of images | 1800 | 1800 |
| Exposure time per image (s) | 0.1 | 0.2 |
| Total exposure time (s) | 180 | 360 |
| Beam size (µm) | 100x100 | 100x100 |
| Flux (photons/sec) | 8.60E+10 | 8.60E+10 |
| **Data reduction and refinement statistics** | | |
| Resolution range | 39.68-0.9 (0.91-0.9) | 39.39-0.9 (0.91-0.9) |
| Space group | P 43 | P 43 |
| Unit cell | 88.72 88.72 39.735 90 90 90 | 88.069 88.069 39.071 90 90 90 |
| Total reflections | 1496597 (41333) | 1454041 (40164) |
| Unique reflections | 227591 (7428) | 220438 (7138) |
| Multiplicity | 6.6 (5.6) | 6.6 (5.6) |
| Completeness (%) | 99.94 (98.72) | 99.88 (97.42) |
| Mean I/sigma(I) | 22.45 (1.91) | 19.26 (1.33) |
| Wilson B-factor | 7.76 | 7.83 |
| R-merge | 0.03619 (0.7356) | 0.04312 (1.084) |
| R-meas | 0.03931 (0.8125) | 0.0468 (1.195) |
| R-pim | 0.01522 (0.3385) | 0.01803 (0.4937) |
| CC1/2 | 1 (0.753) | 1 (0.602) |
| CC* | 1 (0.927) | 1 (0.867) |
| Reflections used in refinement | 227591 (7428) | 220438 (7138) |
| Reflections used for R-free | 11057 (372) | 10729 (346) |
| R-work | 0.1117 (0.2314) | 0.1143 (0.2625) |
| R-free | 0.1257 (0.2382) | 0.1233 (0.2976) |
| CC(work) | 0.976 (0.896) | 0.979 (0.844) |
| CC(free) | 0.973 (0.921) | 0.979 (0.834) |
| Number of non-hydrogen atoms | 3444 | 3322 |
| macromolecules | 2724 | 2772 |
| ligands | 0 | 36 |
| solvent | 720 | 514 |
| Protein residues | 338 | 338 |
| RMS(bonds) | 0.004 | 0.019 |
| RMS(angles) | 0.82 | 0.85 |
| Ramachandran favored (%) | 100 | 99.1 |
| Ramachandran allowed (%) | 0 | 0.6 |
| Ramachandran outliers (%) | 0 | 0.3 |
| Rotamer outliers (%) | 0.99 | 0.32 |
| Clashscore | 3.1 | 3.71 |
| Average B-factor | 13.01 | 12.68 |
| macromolecules | 9.86 | 10.22 |
| ligands |  | 7.05 |
| solvent | 24.93 | 26.35 |
