# Supplementary material for: A single inactivating amino acid change in the SARS-CoV-2 NSP3 Mac1 domain attenuates viral replication in vivo
Source: PLoS Pathog. 2023 Aug 31;19(8):e1011614. doi: 10.1371/journal.ppat.1011614 (PMC10499221; doi:10.1371/journal.ppat.1011614)
Supplement: Attachment — Submitted filename: Mac1 PLoSPath rebuttal_V4.docx [file ppat.1011614.s013.docx]

We thank the reviewers for their thorough review and valuable comments regarding our manuscript. We are grateful that the reviewers recognized our study as a “novel” and “important, well-written and timely paper” that provides “new facets of Mac1 domain in virus replication.” Two broader issues were raised that relate to the presentation and interpretation of the data as well as to the central question whether the Mac1 activity is directly affecting viral replication vs antagonizing host IFN responses. We have addressed these by adding new data to the revised manuscript and by improving the description and clarifying the interpretation of the data. Below are point-by-point responses to the comments raised by the reviewers.

Reviewer #1: Major concerns:
1 The authors claimed that "The N40D mutant generated similar luciferase signals to the WT replicon in both VAT and Calu3 cells... (Fig. 2B).", as well as "This mutation, when introduced via reverse genetics into fully infectious viral clone or replicons, showed minimal effects on viral replication in immortalized cell lines while modestly inducing the IFN response". However, in Figure 2B, the authors showed significant difference in RLU signals between WA1 and WA1 Mac1 N40D (p<0.001), which can not be treated as "similar" or "mininal" and may affect the conclusion drawn from these data. In addition, the authors are strongly recommended to repeat these assays by using rescued virus carrying point mutations (especially N40D).

We thank the reviewer for pointing this out. We have revised the text to indicate that the differences in luciferase signal in VAT cells but not Calu3 cells are significant (page 5 line 186). Of note, quantification of the RNA levels by RT-qPCR in Calu3 cells showed no significant difference between the WT and N40D mutant replicons (Fig. 2C). We have also conducted these studies with rescued virus carrying the N40D mutation. The N40D mutant virus produced 2-fold lower number of particles and similar intracellular viral RNA (Fig. 3B and 3C). During the review period, we have repeated these studies as requested and show that particle production was not significantly different between the N40D mutant and WT viruses over 72-hour post-infection period at multiple MOIs, except for increased PFUs produced by the mutant virus at 72 hours post-infection at MOI 0.1 (Suppl Fig. 5, see below).

2 The authors need to be very careful and specific in wording "IFN signal". IFN signals can be comprised of diverse pathways demonstrating distinct functions during SARS-CoV-2 infection and should not be treated equally. In Fig. 2D, the authors showed that WA1 Mac1 N40D infection induced higher IFNb1 but did not affect the expression of STAT1, it is confusing that in subsequnt assays (Fig. 2E), the authors used IFN-gamma (more stat1-related) instead of IFN-beta to treat infection system. These assays need to be justified.

We apologize for the confusion. We have conducted the experiment in Fig. 2E (see below) also with IFN-beta, which is now included as Supplemental Fig. 4 (see below) and discussed in the text on page 5 line 200. We do not observe a difference between the WT and N40D mutant virus after IFN-beta treatment, which is consistent with a recent preprint showing no difference between wild-type and Mac1 deletion mutant SARS-CoV-2 after treatment with IFN-beta but a significant difference when the cells were treated with IFN-gamma (see ref 34 in the paper and Fig. 3 from the reference below). IFN-gamma has reduced SARS-CoV-2 antiviral activity and induces a restricted subset of ISGs compared with IFN-beta (Busnadiego et al. mBio. 2020). We hypothesize that the overall stronger inhibition of IFN-beta compared with IFN-gamma on SARS-CoV-2 replication may explain why we don’t see a difference between WT and mutant virus in infected cells treated with IFN-beta.

3 The authors claimed that "at 48 hours after infection, particle production was measured by plaque assays while intracellular viral RNA and cytokine expression levels were measured by RT-qPCR. The N40D Mac1 mutant showed modestly (~2-fold) lower particle production and intracellular viral RNA compared with the WT virus suggesting a slightly attenuated phenotype (Fig. 2C and 2D). Similar to the results from replicon experiments, the N40D mutant induced higher levels of IFN as compared to WT virus (Fig. 3D)", but did not showed any statistics outcome in corresponding figures.

We have updated the top portion of the figure to include statistical tests between the WA1 and N40D mutant virus in Fig. 2D and 3B-D and revised the text to indicate where the differences were significant or not significant on page 5 and line 220 as requested.

4 The authors interpreted data shown in Fig. 3F as "loss of the IFN antagonism
limits viral infection by the mutant virus, especially at later time points after infection, and differs from previous work with SARS-CoV where the Mac1-deficient virus phenotype is apparent early post infection". The explanation is very confusing. In addition, the decrease of virus titer at 48 and 72 hours could not be attributed to less efficient infection. Instead, it showed the accelerated virus clearance to me.

We agree and have revised the statement to propose that in contrast to SARS-CoV where the difference in replication between WT and mutant virus is associated with a defect in viral fitness (i.e. as seen early post infection, see below from Kuri et al, Ref 33, Fig. 1D), in SARS-CoV-2, the difference is related to enhanced viral clearance (i.e. as seen late post infection, see below this study Fig. 3F and discussed on page 6 and	233).

5 The authors need to have independent pathologists check and score the lung pathogenic changes shown in Fig. 4G.

We have now included mouse lung pathology analysis by independent pathologists in Fig. 4H and Supplemental Fig. 7 and discussed on page 6 and line 260. The subpanels in 4G have also been updated with representative lung sections. This shows that both the wild-type and mutant viruses cause lung immune pathology to a similar extent. The significant reduction in viral titer for the mutant virus but similar lung immune pathology underscores the model that a defect in antagonism of host immune responses and faster viral clearance of the mutant virus is responsible for reduced viral titers rather than a defect in infectivity. We added discussion of how these results differ from a recent preprint (Alhammad et al, Ref 34) with a Mac1 deletion mutant SARS-CoV-2 that shows reduced lung pathology. We suggest that this discrepancy is seen because the deletion mutant shows lower viral titers at all times post-infection, likely indicating a defect in viral fitness independent from the Mac1 catalytic activity (Alhammad et al, Fig. 5E and 5F, see below). In contrast and as discussed, the point mutant reported in our study that only abolishes Mac1 catalytic activity shows decreased viral titers only at late time points while replicating even better than WT at early time points, supporting the model that it is cleared faster than the wild-type virus due to a vigorous immune response that causes the observed lung immune pathology (this study, Fig 4E and 4F below).

Reviewer #2: The in vitro data in its current state does not robustly support claims that the host antiviral response is the mechanism for the attenuation of N90D. Loss of NSP3 Mac 1 catalytic activity could be impacting viral replication directly.
As reviewer #1 points out, the characteristic kinetics of the mutant virus replication underscore the model that viral clearance rather than viral replication is affected by the mutation. This is supported by the organoid and in vivo data where the mutant virus infects and replicates as well (organoids) or better (mice) than the WT virus early post infection and shows decreased viral titers only at late time points (See Fig. 3F and 4E below). In addition, no effect was observed in replicon experiments where the infection is limited to a single round. We have clarified that we propose a model where the attenuation of the mutant virus is due to faster viral clearance as opposed to an inherent replication defect. This is now included on page 6 line 231 and on page 6 line 266.

1. Please clarify whether equivalent infectious doses used to infect cells with replicon and recombinant viruses. What is the proportion of cells that is infected/transduced by WT and N40D viruses? Does decreasing the infectious dose enhance the detection of the replication defect of the catalytic mutant?
We carefully equalize amounts of plasmids that were transfected for the replicon experiments and infectious doses of supernatants that were utilized for the infection experiments. For the rescued viruses, equal infectious doses of the WT and N40D mutant viruses (as measured by plaque assays) were utilized in all experiments. In the replicon experiment, we typically observe 40–50% of cells to be EGFP+. Interestingly, decreasing the infectious dose does not increase detection of the difference in viral titers between WT and mutant virus as shown in new data performed in Calu3 cells with 0.01 and 0.1 MOI, where we observe similar results to what is shown in Fig. 3, even detecting inverse results for WT and mutant virus at 72 hours at MOI 0.1. These additional data are included in Supplemental fig. 5 (see below) and discussed in the text on page 5 line 218 and underscore the model that no inherent replication defect is observed for the mutant virus.

2. In figure 2E the authors treat cells with IFN gamma to determine whether the cell intrinsic IFN induction could suppress the N40D mutant. IFN gamma is not synthesized by infected Calu3 cells, rather viral infection induces the expression of type I and/or type III IFNs. Thus, it would be more appropriate to contrast cell intrinsic IFN sensitivity to conclude that Mac1 is required to overcome antiviral immunity. Furthermore, the authors conclude that IFN gamma does not hinder WT infection at either 10or100ng/ml concentration, yet there is a two-log decrease N copy number. Have statistics been run on these data? Is this change significant given that there is only a ~2 fold change between mutants across doses? While this is cited in the figure legends, there is no indication of significance on the figure itself. If so, a Student’s t test is not an appropriate statistic for these comparisons (effect mutants and effects doses). A better way to determine whether the host antiviral response contributes to viral attenuation of N90D would be to infect IRF3-deficient cells and determine whether the 2-fold attenuation is conserved.

We apologize that the description of the experiment was not clear. We have revised the statement on page 5 line 200 to indicate that the comparisons were made with regards to the mutant and WT virus and not the different doses of IFN-gamma to the no-treatment control. In addition, the Figure was updated to indicate that the difference was not statistically significant. We have conducted the experiment in Fig. 2E (see below) also with IFN-beta, which is now included as Supplemental Fig. 4 (see below) and discussed in the text on page 5 line 203. We do not observe a difference between the WT and N40D mutant virus after IFN-beta treatment, which is consistent with a recent preprint showing no difference between wild-type and Mac1 deletion mutant SARS-CoV-2 after treatment with IFN-beta but a significant difference when the cells were treated with IFN-gamma (see ref 34 in the paper and Fig. 3 from the reference below). IFN-gamma has reduced SARS-CoV-2 antiviral activity and induces a restricted subset of ISGs compared with IFN-beta (Busnadiego et al. mBio. 2020). We consider that the overall stronger inhibition of IFN-beta compared with IFN-gamma on SARS-CoV-2 replication may explain this lack of difference.

3. As the overall hypothesis of this study is that the host innate immune response is most competent in primary tissues, it is important to demonstrate that decreases in viral titers of Mac1 mutants is also concordant with increased antiviral and inflammatory responses in human organoids (Fig 3F). Are these changes more robust than the modest changes in gene expression seen in Calu3 cells? As the authors conclude that “It is possible that the modest phenotype of the N40D mutant observed in Calu3 cells is due to these cells not fully recapitulating the cellular environment during SARS-CoV-2 infection” and go on to further expand upon this concept in the discussion, the production of infectious virus should be measured at 72hrs post infection. The differences in infectious virus production in Calu-3 were similar to those observed at 48hrs in organoids. The sample size should also be increased in order to determine whether any of the changes in infectious virus production or gene expression are significant between viruses.

We conducted the requested analyses (new Fig. 3G, see below) and found that indeed, organoids infected with the mutant virus had higher expression of several innate immune response genes compared with organoids infected with the wild-type virus, including ISG15, IFNb, IL6, MX1, and IFNa. Interestingly, the magnitude of differences in innate immune response gene induction between the WT and mutant virus was mostly similar to that observed in Calu3 cells (Fig. 3C, see below), suggesting a potential downstream defect in viral clearance in Calu3 cells but not organoids. With regards to the levels of induction between Calu3 cells and organoids, ISG15 and IFNb were higher in Calu3 cells while IL6 was higher in organoids.

Additionally, we measured infectious particle production in Calu3 cells over 24, 48, and 72 hours post infection as requested and at different MOI as requested, which is now included in Supplemental Fig. 4 (see below) and discussed on page 5 Line 218.

Reviewer #3: None.

Reviewer #4: No major issues noted on the conduct of the studies in the analysis of the role of N40D. The data are more relevant to basic understanding of Mac1 then to approaches for therapy. The investigators might want to change the Discussion to address this point.

We agree and have now modified the discussion to focus on the basic understanding of Mac1 rather than therapy.

**Part III – Minor Issues: Editorial and Data Presentation Modifications**
Please use this section for editorial suggestions as well as relatively minor modifications of existing data that would enhance clarity.

Reviewer #1: Minor concerns
The authors need to double-check the typos and figure indicators.
In "The N40D Mac1 mutant showed modestly (~2-fold) lower particle production and intracellular viral RNA compared with the WT virus suggesting a slightly attenuated phenotype (Fig. 2C and 2D).", the correlating figures should be 3B and 3C.
In "While WT and Mac1 N40D mutant viruses produced similar levels of infectious particles at 24 hours post infection, the Mac1 N40D mutant showed ~10-fold lower particle production at 48 and 72 hours post infection (Fig. 4B).", the correlating figures should be 3F.
Finally, the authors are suggested to re-write or divide some long sentences and make the points clearer.

We thank the reviewer for pointing these out and they have corrected them in the revised manuscript.

Reviewer #2: Additional editing of the manuscript and statistical analysis is necessary to better understand experimental design and data interpretation.

1. The authors conclude in Figure 2B that the relative luciferase signals of WT and N90D mutant are similar, yet the statistical analysis suggest differences in the RLU between these two groups. The authors should clarify whether a modest decrease in infectious virus packaging was observed.

We agree and have revised the text to indicate the differences on page 5 line 186. Infectious particle production was measured in Calu3 cells and the differences between the WT and N40D mutant viruses were, indeed, statistically significant (Fig. 3B).

2. The authors identify IFNB1, IL-6, STAT1 and ISG15 as cytokines. Only IFNB1 and IL6 fit that description and STAT1 and ISG15 are best described as ISGs. Similarly in the date presented in Fig 4. MX1 is not a cytokine. Consider updating this.

We have modified the terminology as suggested to “innate immune response genes” to address this point in the revised manuscript.

3. Could the authors clarify the methodology for quantifying the relative expression of host innate immune genes across figures 2and 3? Are the changes in gene expression conserved when contrasting to a house keeping gene rather than a spike control? There could be differential cell death across mutants resulting in changes in gene detection.

In both Figures the host innate immune genes were measured relative to GAPDH housekeeping control. In Fig. 2, the data was further normalized to the “-Spike control” of each replicon. The -Spike control contains particles with a packaged replicon genome but will not have Spike on the surface. We utilized this control to account for any replicon specific background induction of host innate immune responses by these particles. We have clarified this now in the text and Figure legend.

4. The authors should revise the figure callouts and legends. Figure legend labels for Figure 4G and 4H are swapped. Refer to figures 3B and 3C in main text. Figure 3F is called out as figure 4B in main text.

We thank the reviewer for pointing these out. This has been corrected in the revised manuscript.

5. Could the authors please clarify what is mean by the statement: “underscoring the specific effect of this mutation in the viral life cycle.” Is the specific effect innate immune activation?

We have revised this statement to “underscoring the potential role of Mac1 catalytic activity in innate immune response antagonism.”

6. How many animals does the data presented in figure correspond to for each infectious group (days post infection)? 5 each?

Yes, this is now indicated in the methods section of the manuscript and now has been added to the figure legend of Fig. 4 in the revised manuscript.

7. For figure 4G consider highlighting the area of the lung that was magnified and including images from replicate lungs. Quantification of lung pathology would increase rigor. Does N90D infect areas of the lung distinct from those targeted by WA-1?

We have highlighted the magnified area and updated the figure in the revised manuscript with representative lung histology. In addition, we have now included pathology analysis by independent pathologists in Fig. 4H and Supplemental fig. 7 and discussed on page 6 and line 260. We find that both the WT and mutant viruses cause lung immune pathology to a similar extent. This underscores the model that the mutant virus is cleared faster due to a vigorous immune response rather than reduced replication capacity. We did not identify areas of the lung differentially infected by the mutant virus.

Reviewer #3: Minor comments:
Introduction – ‘Specifically, PARP7, PARP9, PARP12 and PARP14 directly enhance antiviral innate immune responses (24-26) and are upregulated during MHV infection (27).’ – I don’t think PARP7 enhances antiviral responses, it’s quite the opposite.

We thank the reviewer for pointing this out. This has been addressed in the revised manuscript to indicate that PARP7 has pro- and anti-viral roles on page 3 and Line 95.

Introduction – it would be very good to clearly mention that PARP14 is the main antiviral PARP acting against coronaviruses and that Mac1 reverses PARP14-mediated ADP-ribosylation (doi: 10.1371/journal.ppat.1007756; doi: 10.1098/rsob.200237).

This has now been added to the introduction with indicated citations on page 3 Line 96.

Supplementary Figure 1 – it would be good to label the catalytic residue in the figure.

N40 has been labeled in the revised Supplemental Figure 1.

Discussion – considering that the presented results greatly motivate further efforts in development of Mac1 inhibitors, it would be good to discuss more extensively the current literature describing attempts to develop such inhibitors.

We have now included a brief discussion of the current efforts to develop Mac1 inhibitors in the revised manuscript on page 7 Line 336.

Reviewer #4: Minor proofreading:
1. Introduction, paragraph 3, line 1, correct spelling of ADP-ribosylhydrolase not -layse.
2. In figure 1, a graph from figure 1H is present, but it is not discussed in text and within legend.
3. In figure 4, Figure 4 G and figure 4H, need to be switched from what is actually in the figure.

We thank the reviewer for pointing these out. All have been corrected in the revised manuscript.
